# PuraStat as secondary therapy for hemostasis in Mallory−Weiss syndrome with oral antithrombotic medication

**DOI:** 10.1002/deo2.70033

**Published:** 2024-11-19

**Authors:** Makoto Higashino, Hidehiro Murakami, Tetsu Hirata, Hiroaki Miyaoka

**Affiliations:** ^1^ Department of Internal Medicine Saiseikai Matsuyama Hospital Ehime Japan; ^2^ Department of Internal Medicine Ozu City Hospital Ehime Japan

**Keywords:** endoscopic hemostasis, esophagus, gastrointestinal bleeding, Mallory–Weiss syndrome, PuraStat

## Abstract

Mallory−Weiss syndrome (MWS) is a common cause of gastroesophageal bleeding. Vomiting increases intra‐abdominal and intra‐esophageal pressures, causing hyperextension of the esophagogastric junction and laceration. Most affected patients respond well to conservative treatment; however, those with active bleeding require endoscopic intervention. Upon contacting blood, PuraStat gels and coats the bleeding point to achieve hemostasis. PuraStat is reportedly effective for non‐variceal bleeding and bleeding associated with endoscopic procedures. However, there have been no reports on the use of PuraStat in MWS. Here we report a case in which PuraStat was useful for achieving hemostasis in a patient with MWS and difficult‐to‐achieve hemostasis. The patient was a 67‐year‐old man who had undergone coronary artery bypass grafting 1 month earlier and was taking an antithrombotic drug. He visited our hospital with bloody vomiting and melena in the evening and was diagnosed with upper gastrointestinal bleeding for which he underwent endoscopy. MWS with active bleeding was observed in the lower esophagus extending to the esophagogastric junction. We treated the patient with clipping; however, the oozing did not stop because of the large laceration. We applied PuraStat to the bleeding site and confirmed that the oozing had resolved; therefore, the procedure was terminated. The endoscope was reinserted the next day and confirmed the hemostasis. The patient was discharged without further deterioration. In patients with MWS with active bleeding, endoscopic hemostasis is commonly achieved using clips or endoscopic band ligation. However, PuraStat can achieve complete hemostasis when these techniques fail.

## INTRODUCTION

Mallory−Weiss syndrome (MWS) is caused by increased intra‐abdominal and intra‐esophageal pressure due to vomiting that hyperextends the esophagogastric junction and causes lacerations. MWS accounts for 5–15% of cases of upper gastrointestinal bleeding,^1^ with most cases improving with conservative treatment; however, active bleeding requires endoscopic treatment.^2^ Endoscopic treatments include endoscopic hemoclip placement (EHP)^3^ and endoscopic band ligation (EBL).^4^

PuraStat (3‐D Matrix) is composed of three repeating amino acids – arginine, alanine, and aspartic acid – that gelatinize to form a three‐dimensional matrix upon exposure to blood. The gelatinized PuraStat physically coats the bleeding point to achieve hemostasis. PuraStat has been used in gastrointestinal endoscopy for non‐variceal gastrointestinal bleeding and bleeding associated with endoscopic procedures.^5^

In our case, hemostasis was successfully achieved using a PuraStat after EHP, which may increase options for the hemostatic treatment of MWS.

### Case report

**FIGURE 1:**
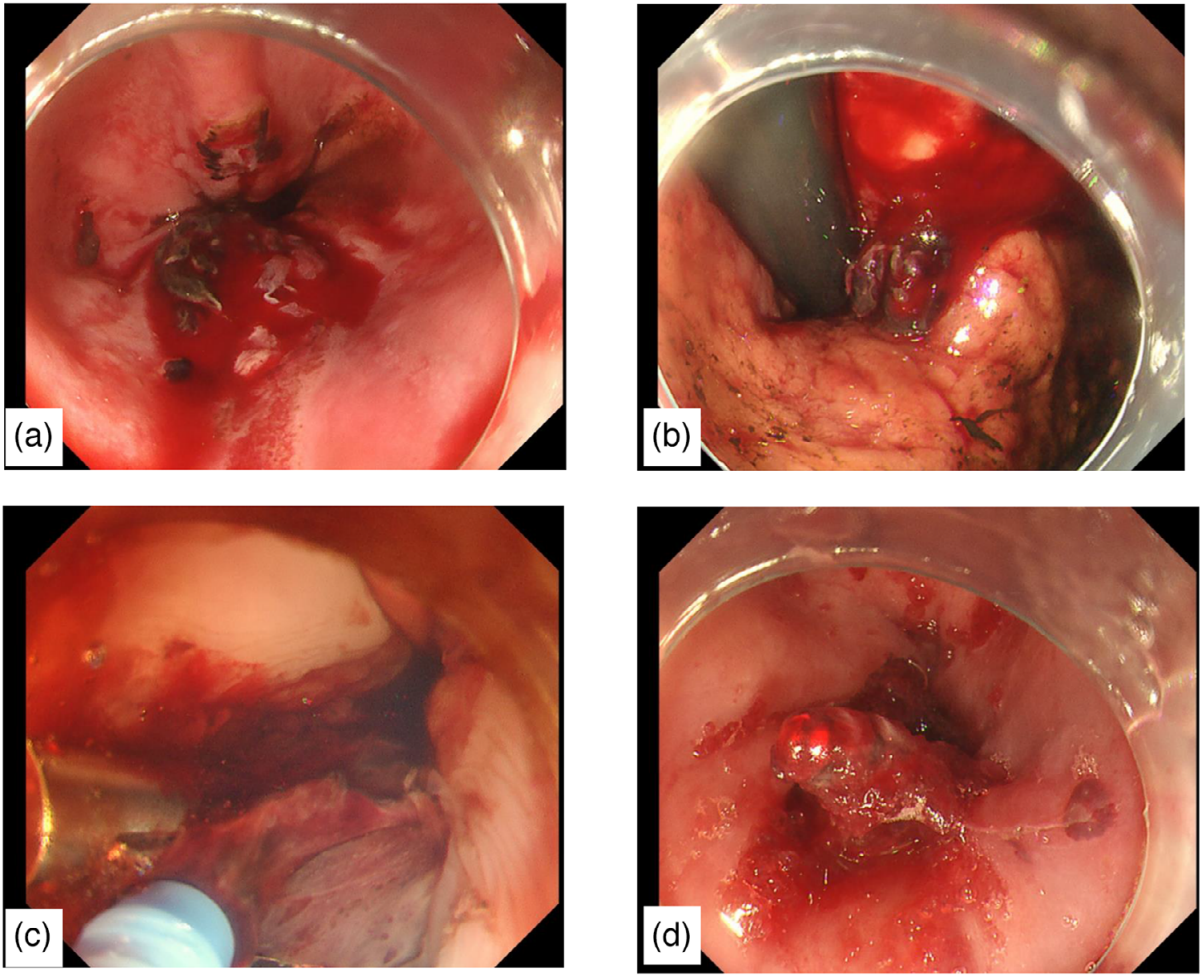
Emergency gastrointestinal endoscopy. (a) A large actively bleeding laceration is visible in the lower esophagus. (b) The laceration reached the cardia. (c) As clipping of the laceration wound did not achieve hemostasis, PuraStat was applied. (d) Hemostasis after PuraStat application.

A 67‐year‐old man started vomiting in the morning, and the vomit gradually turned bloody. The patient was admitted to our hospital in the evening with melena and lightheadedness. He had a medical history of type 2 diabetes mellitus, hypertension, and coronary artery disease for which he had undergone coronary artery bypass grafting at another hospital approximately 1 month before presentation, where he was prescribed clopidogrel. On arriving at our hospital, his vital signs were blood pressure of 131/82 mmHg, pulse of 103/min, temperature of 37.0°C, and O_2_ saturation of 98% on room air. A physical examination revealed no conjunctival pallor and his abdomen was flat, soft, and tender. A rectal examination revealed no palpable masses; however, black stools were observed. A laboratory evaluation showed anemia with a hemoglobin of 10.1 g/dL and elevated blood urea nitrogen (BUN)/creatinine (Cr) ratio: BUN 44.4 mg/dL, Cr 0.96 mg/dL (Table 1). Non‐contrast computed tomography of the abdomen revealed no findings suggestive of gastrointestinal perforation. Upper gastrointestinal bleeding was diagnosed, and an emergency esophagogastroduodenoscopy was performed (Figure 1). Multiple lacerations with Forrest classification Ib hemorrhage extending from the lower esophagus to the esophagogastric junction were diagnosed as MWS.

**TABLE 1:** Laboratory data on admission.

Blood count		Biochemistry		Coagulation studies	
WBC	10,110/µL	TP	6.3 g/dL	Prothrombin time ‐ international normalized ratio	1.06
Neut	79.6 %	Alb	3.4 g/dL		
Mono	5.4 %	T‐bil	0.5 mg/dL	Activated partial thromboplastin time	26.5 second
Eosino	0 %	AST	12 IU/L		
Baso	0.1 %	ALT	18 IU/L		
Lymph	14.9 %	γGTP	19 IU/L		
Hb	10.1 g/dL	LDH	127 IU/L		
Ht	30.1 %	CK	44 IU/L		
RBC	334 × 10⁴/µL	BUN	44.4 ng/dL		
PLT	21.2 × 10⁴/µL	Cr	0.96 mg/dL		
		Na	137 mEq/L		
		K	4.4 mEq/L		
		Cl	105 mEq/L		
		CRP	4.37 mg/L		
		BS	160 mg/dl		

Abbreviations: γGTP,γ‐glutamyl transpeptidase; Alb, albumin; ALT, alanine transaminase; AST, aspartate transaminase; Baso, basophil; BS, blood glucose; BUN, blood urea nitrogen; CK, creatine kinase; Cl, chloride; Cr, creatinine; CRP, C‐reactive protein; Eosino, eosinophil; Hb, hemoglobin; Ht, hematocrit; K, potassium; LDH, lactic dehydrogenase; Lymph, lymphocyte; Mono, monocyte; Na, sodium; Neut, neutrophil; PLT, platelet; RBC, red blood cell; T‐bil, total bilirubin; TP, total protein; WBC, white blood cell.

**FIGURE 2:**
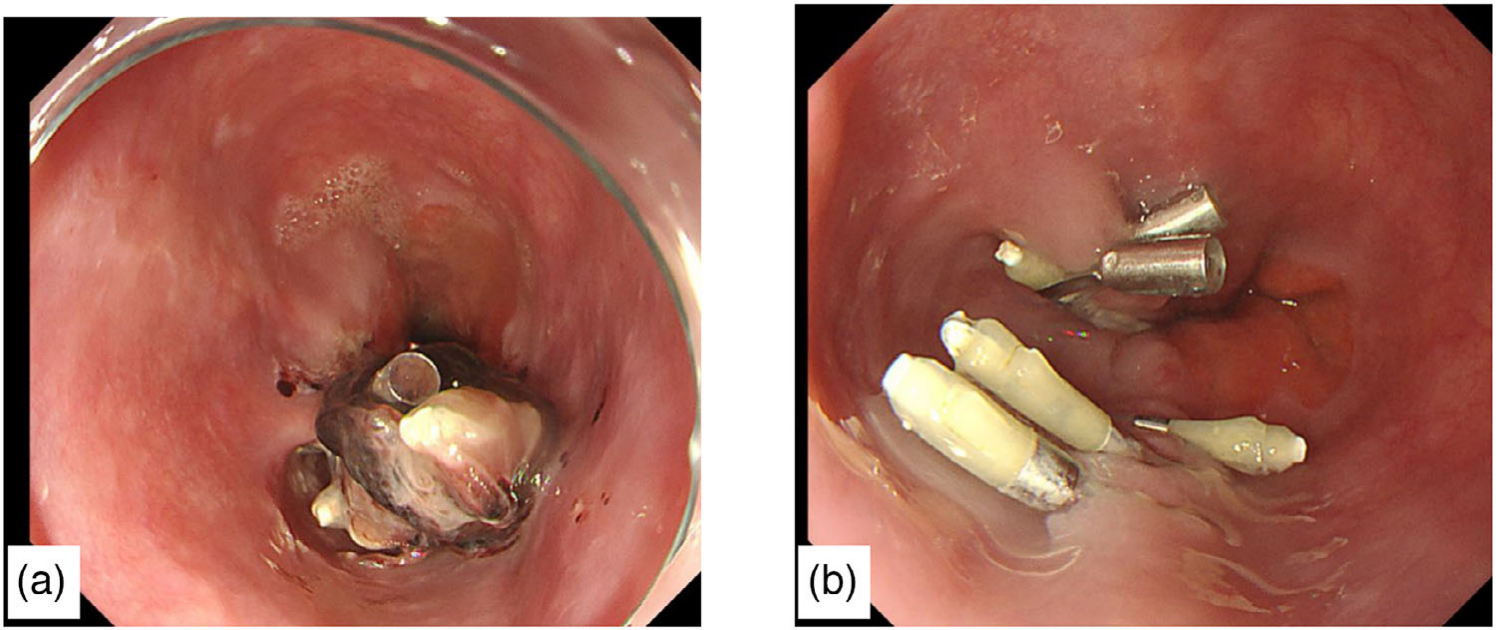
Gastrointestinal endoscopy after treatment. (a) Hemostasis on repeat gastrointestinal endoscopy the day after treatment. (b) Healed laceration wound on gastrointestinal endoscopy on hospitalization day 12.

Considering the thin esophageal wall, we sutured the lesion with a clip rather than cauterizing it. We attempted to suture the laceration from the oral side using clips; however, multiple vomiting episodes during the procedure caused the tear to enlarge and extend in the short‐axis direction. The laceration was located at the 6 o'clock position and the tear and the bleeding sites were obscured by blood, making it difficult to identify the area for clip placement, thus complicating the procedure. Clipping in 14 locations reduced the degree of bleeding but did not stop the oozing. Multiple applications of PuraStat (5 mL) to the bleeding site resolved the oozing. The next day, the endoscopy was repeated and hemostasis was confirmed (Figure 2a), and oral medication including clopidogrel was resumed. Despite a gradual increase in food intake, his symptoms did not worsen. On day 12 of hospitalization, repeated endoscopy confirmed the healing of the lacerated wound (Figure 2b). The patient was discharged on day 13 of hospitalization.

## DISCUSSION

In the present case, complete hemostasis could not be achieved with EHP for active bleeding from MWS; however, it was achieved with secondary therapy using PuraStat. To the best of our knowledge, this is the first report of its kind.

Few studies have compared the efficacy of endoscopic treatments for MWS including EHP and EBL. In a prospective trial comparing 41 patients with MWS and active bleeding who were randomized into EHP and EBL groups, the percentages of patients achieving primary hemostasis were 94% and 90%, respectively, with no significant differences.^6^ Both EHP and EBL have high success rates at achieving hemostasis in MWS.

A systematic review examining the efficacy of PuraStat for treating gastrointestinal bleeding analyzed 17 studies.^7^ Of them, six reported bleeding type, with four involving only oozing and two involving both oozing and spurting. The hemostasis rate was 87.7% (range, 38.1%–100%) and the rebleeding rate was 4.7% (range, 0%–16.2%), which was comparable to or slightly lower than that of other hemostatic techniques. A prospective study by Branchi et al. involving 111 nonvariceal gastrointestinal bleeding cases reported a hemostasis success rate of 94% in 79 cases in which PuraStat was used as the primary treatment.^8^ When used as the secondary treatment in 32 cases, the success rate was 75%. This study included two cases of MWS; however, the details were not specified. Based on these findings, PuraStat appears as effective and safe as conventional hemostatic techniques for the treatment of gastrointestinal bleeding, particularly oozing bleeding. Additionally, it demonstrated high success rates when employed as the secondary treatment in cases in which initial hemostasis was not achieved using other methods. Although the efficacy of PuraStat specifically for MWS has not been thoroughly investigated, its efficacy for other types of gastrointestinal bleeding suggests a potential benefit for MWS as well. Thus, the strategy of initially applying EHP or EBL for MWS‐related bleeding, followed by the addition of PuraStat if hemostasis fails, may prove useful.

PuraStat forms a gel upon contact with blood and covers the bleeding site to achieve hemostasis. In a report by Murakami et al., after a biopsy‐induced hemorrhage in gastric cancer in which EHP failed to achieve hemostasis and subsequent clipping obscured the bleeding site, the application of PuraStat successfully achieved hemostasis.^9^ In situations in which it is difficult to identify the exact bleeding site, the wide application of PuraStat allows coverage of the general area, offering potential hemostatic benefits. In the present case, the active bleeding area was submerged in blood, making visualization of the source challenging after multiple EHP applications. However, the application of PuraStat over a broad area effectively controlled the hemorrhaging.

Although endoscopic hemostasis is achieved using EHP and EBL in many cases of MWS with active bleeding, PuraStat may achieve complete hemostasis when these techniques are insufficient. Further cases should be accumulated to draw definite conclusions regarding the ability of PuraStat to achieve hemostasis in MWS.

## CONFLICT OF INTEREST STATEMENT

None.

## ETHICS STATEMENT

N/A

## PATIENT CONSENT STATEMENT

N/A

## CLINICAL TRIAL REGISTRATION

N/A
